# Towards Deciphering the Fetal Foundation of Normal Cognition and Cognitive Symptoms From Sulcation of the Cortex

**DOI:** 10.3389/fnana.2021.712862

**Published:** 2021-09-28

**Authors:** Arnaud Cachia, Grégoire Borst, Renaud Jardri, Armin Raznahan, Graham K. Murray, Jean-François Mangin, Marion Plaze

**Affiliations:** ^1^Université de Paris, LaPsyDÉ, CNRS, Paris, France; ^2^Université de Paris, IPNP, INSERM, Paris, France; ^3^Institut Universitaire de France, Paris, France; ^4^Univ Lille, INSERM U-1172, CHU Lille, Lille Neuroscience & Cognition Centre, Plasticity & SubjectivitY (PSY) team, Lille, France; ^5^Section on Developmental Neurogenomics, Human Genetics Branch, National Institute of Mental Health, Bethesda, MD, United States; ^6^Department of Psychiatry, University of Cambridge, Cambridge, United Kingdom; ^7^Université Paris-Saclay, CEA, CNRS, Neurospin, Baobab, Gif-sur-Yvette, France; ^8^GHU PARIS Psychiatrie & Neurosciences, site Sainte-Anne, Service Hospitalo-Universitaire, Pôle Hospitalo-Universitaire Paris, Paris, France

**Keywords:** sulcation, gyrification, neurodevelopment, MRI, psychology, psychiatry

## Abstract

Growing evidence supports that prenatal processes play an important role for cognitive ability in normal and clinical conditions. In this context, several neuroimaging studies searched for features in postnatal life that could serve as a proxy for earlier developmental events. A very interesting candidate is the sulcal, or sulco-gyral, patterns, macroscopic features of the cortex anatomy related to the fold topology—e.g., continuous vs. interrupted/broken fold, present vs. absent fold-or their spatial organization. Indeed, as opposed to quantitative features of the cortical sheet (e.g., thickness, surface area or curvature) taking decades to reach the levels measured in adult, the qualitative sulcal patterns are mainly determined before birth and stable across the lifespan. The sulcal patterns therefore offer a window on the fetal constraints on specific brain areas on cognitive abilities and clinical symptoms that manifest later in life. After a global review of the cerebral cortex sulcation, its mechanisms, its ontogenesis along with methodological issues on how to measure the sulcal patterns, we present a selection of studies illustrating that analysis of the sulcal patterns can provide information on prenatal dispositions to cognition (with a focus on cognitive control and academic abilities) and cognitive symptoms (with a focus on schizophrenia and bipolar disorders). Finally, perspectives of sulcal studies are discussed.

## Introduction

**Figure 1:**
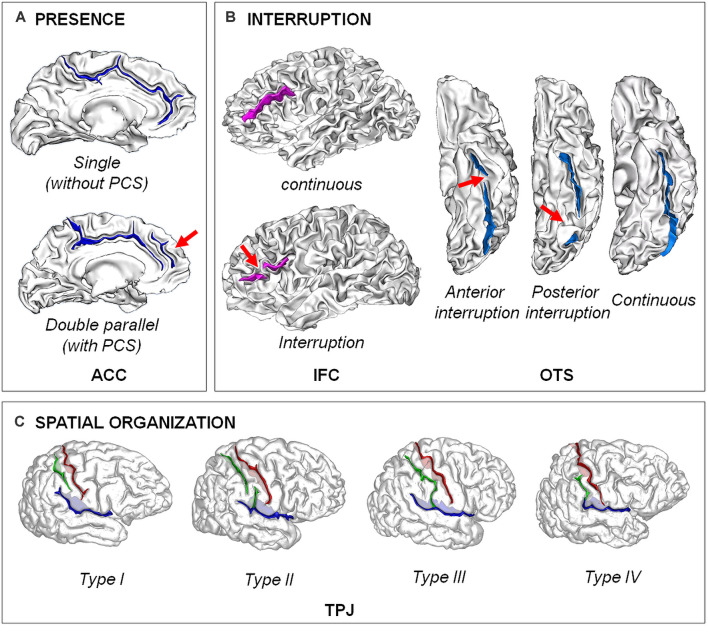
Examples of different types of sulcal patterns. **(A)** Presence of the fold. The sulcal pattern of the anterior cingulate cortex (ACC) can be “single”, with only the cingulate sulcus, or “double parallel”, with an additional paracingulate sulcus (PCS). Adapted from Cachia et al. (2014). **(B)** Interruption of the fold. The sulcal pattern of the inferior frontal cortex (IFC) can be continuous or with an interruption (red arrow). The sulcal pattern of the lateral occipito-temporal sulcus (OTS) can be continuous or have an anterior or a posterior sulcal interruption. Adapted from Cachia et al. (2014); Borst et al. (2016); Cachia et al. (2018), and Tissier et al. (2018). **(C)** Spatial organization. The sulcal pattern of the temporo-parietal junction (TPJ) is characterized by the spatial organization of the posterior part of the right Sylvian fissure (pSF) including Sylvian fissure (in blue), post-central sulcus (green) and central sulcus (in red). Type I, has both a vertical branch (planum parietale) that ascends into the supramarginal gyrus and a horizontal branch (planum temporale) that forms the superior surface of the superior temporal gyrus. In Type II, the Sylvian fissure lacks a vertical branch. In Type III, the horizontal branch extends posteriorly to the supramarginal gyrus into the angular gyrus. In Type IV, the vertical branch connects the post-central sulcus anterior to the supramarginal gyrus, the horizontal branch is therefore absent. Adapted from Steinmetz et al. (1990) and Plaze et al. (2015).

**Figure 2:**
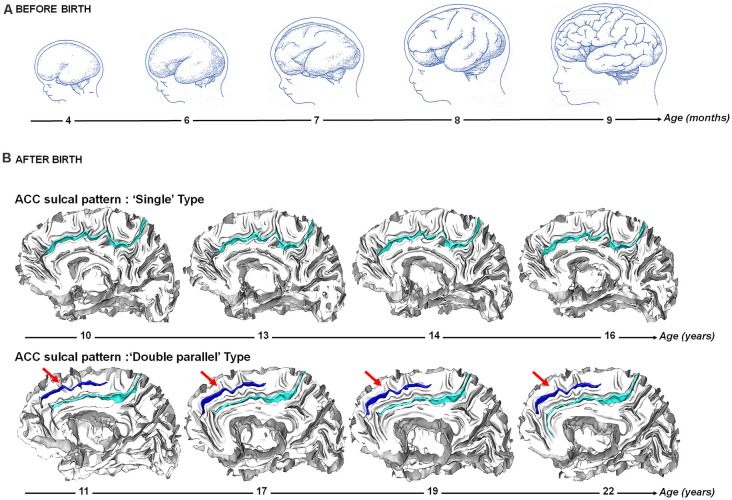
Sulcogenesis before and after birth. **(A)** Before birth. Cortical folding during from 4–9 months post-conception during fetal life. Adapted from Welker (1990). **(B)** After birth. Longitudinal stability of the sulcal pattern of the Anterior Cingulate Cortex (ACC) pattern from 10–22 years in a participant with a “single type” ACC and in a participant with a “double parallel type” ACC. Adapted from Cachia et al. (2016).

Analysis of the brain structure from *in vivo* magnetic resonance imaging (MRI) is now a main tool in biological psychology and psychiatry. Cognitive abilities and psychiatric disorders have been associated to variations in various structural brain features in postnatal life (Giedd and Rapoport, 2010; Kanai and Rees, 2011). However, growing evidence suggest that prenatal processes play a critical role for cognitive ability (Shenkin et al., 2004; Raznahan et al., 2012; Walhovd et al., 2012) and disease risk (Schlotz and Phillips, 2009) that appear in postnatal life (Schork et al., 2019). Such findings have driven the search for anatomical brain features in postnatal life that could serve as a proxy for fetal events. A very interesting candidate is the typologies of cortical folding, also referred as sulcal/sulco-gyral patterns (Figure 1), related to the fold topology—e.g., continuous vs. interrupted/broken fold (Cachia et al., 2018), present vs. absent fold (Yücel et al., 2001)—or their spatial organization—e.g., “H-shaped” sulcus (Nakamura et al., 2007) or “power button-shaped” (Mellerio et al., 2015). Indeed, as opposed to local *quantitative* features of the cortical sheet (e.g., thickness, surface area or curvature/gyrification) taking decades to reach the levels measured in adults (Armstrong et al., 1995; White et al., 2010; Raznahan et al., 2011; Li et al., 2014), the *qualitative* regional pattern derived from the primary, secondary and tertiary folds, or sulci, observed in adults is already evident at birth and stable during the development (Chi et al., 1977; Cachia et al., 2016; Tissier et al., 2018; Figure 2). The analysis of such trait features of the brain can thus give information on the prenatal constraints imposed by some specific brain regions on later cognitive development.

In this review article, we show in some compelling examples that the analysis of the cortex sulcal patterns can help deciphering the fetal foundation of normal cognition in non-clinical populations and cognitive symptoms in patients with psychiatric disorders. After a general overview of the cerebral cortex sulcation mechanisms—indicating why, how and when the cerebral cortex folds along with methodological issues on how to measure the cerebral cortex folds—we present studies illustrating that analysis of the sulcal patterns can provide relevant information on prenatal dispositions to cognition (with a focus on cognitive control and academic abilities) and cognitive symptoms (with a focus on schizophrenia and bipolar disorders). Finally, the limits and perspectives of sulcal studies are discussed.

## Cerebral Cortex Sulcation

### What Is Cortical Folding?

Cortical folding is a characteristic of many mammalian brains, which is intrinsically related to the organization of the brain function (Welker, 1988). The degree of folding increases with the size of the brain (Zilles et al., 2013). This degree of folding scales uniformly as a function of the product of the cortical surface area (CSA) and the square root of cortical thickness (CT; Mota and Herculano-Houzel, 2015). In humans, sulci emerge in a specific order during the perinatal period, with the primary involutions appearing in the second trimester, followed by important growth of folds in the third trimester (Chi et al., 1977). The sulcal pattern observed after birth is the consequence of pre-natal and peri-natal processes that shape the cortex from a smooth surface to a highly convoluted structure (Haukvik et al., 2012; Nishikuni and Ribas, 2013).

The folding process might enable increases in cortical surface area while limiting accompanying increases in axonal wiring costs (Klyachko and Stevens, 2003). This association between network functioning and cortical folding may mediate relationships between cortical folding and brain function. Macroscopic (morphological/volumetric) and microscopic (cellular) features of the cortex are therefore intrinsically interrelated as shown in the early brain mapping studies at the beginning of the 20th century (Brodmann, 1909). For instance, it has been shown that the cortical ribbon is thicker in the gyral areas and thinner in the sulcal areas and neurons located in the deep layers of gyri are squeezed from the sides and appear elongated while neurons that reside in the deep layers of sulci are stretched and look flattened (Hilgetag and Barbas, 2006, 2009). In addition, gyral and sulcal architecture is intrinsically related to the functional organization of the brain. For instance, the central sulcus separates the somatosensory cortex from the motor cortex, the calcarine sulcus separates the superior and inferior visual hemifields and even in the very complex and variable prefrontal cortices, the cortex sulcation influences the functional organization (Li et al., 2015; Lopez-Persem et al., 2019). In addition, functional differences (time-frequency activity, connectivity) exist between sulcal and gyral areas (Jiang et al., 2021).

### When Do the Cortical Sulci Appear?

Human cortex development is a complex and dynamic process that begins during the first weeks of pregnancy and lasts until early adulthood (Figure 2). In parallel to cellular changes, early cortex development is characterized by dramatic changes in its macroscopic morphology due to the cortical folding process that begins from 10 weeks of fetal life (Feess-Higgins and Larroche, 1987; Nishikuni and Ribas, 2013). During the third trimester of pregnancy, the cerebral cortex changes from a relatively smooth, lissencephalic surface to a complex folded structure that closely resembles the morphology of the adult cortex. The development of folds is relatively conserved across individuals and species: primary sulci, which develop first, are the less variable and most heritable (Lohmann et al., 1999) folds, and have the strongest relationship with cytoarchitecture (Welker, 1990; Fischl et al., 2008). Some folding patterns are preserved across species, complex patterns in larger brains likely emerging from simplified patterns in smaller brains (Borrell and Reillo, 2012). The heritability of cortical folding is estimated between 20 and 50% (Le Guen et al., 2018; Pizzagalli et al., 2020), supporting a major effects of early environmental factors on sulcation and cognition, including alcohol exposure (De Guio et al., 2014), intrauterine growth restriction (Dubois et al., 2008a) or twin pregnancy (Amiez et al., 2018).

Dedicated MRI acquisition and morphometric tools have recently allowed to map the developing cortical surface and growth patterns in fetuses as young as 20 weeks of gestational age (Habas et al., 2012). These *in vivo* studies confirm earlier *post-mortem* data (Chi et al., 1977) and show that the cortical folds emerge in a specific order during the prenatal life: stable primary folds appear around 20 weeks of gestational age, secondary folds around 32 weeks of gestational age and highly variable tertiary folds around term (Chi et al., 1977). Gyrification (the emergence/appearance of gyri, the “mountains” of the cortical relief) and sulcation (the emergence/appearance of sulci, the “valleys” of the cortical relief) become manifest after 24 weeks of gestational age (Rajagopalan et al., 2011), and continue to develop during the last weeks before birth (Dubois et al., 2008b, 2019). Although some inter-individual variability is observed, the regional pattern is relatively stable over the brain surface: sulcation starts in the central region with a first wave towards the temporal, parietal and occipital lobes, and a second wave towards the frontal lobe (Ruoss et al., 2001; Dubois et al., 2008b). In contrast to the primary sulci that highly consistently express across (nearly) all humans, certain secondary sulci can vary considerably between individuals (Figure 1). At birth, the area of the cortical surface is three times smaller than in adults, but the cortex is similarly folded and the most variable sulci are the same in newborns and adults (Hill et al., 2010). Longitudinal studies after birth indicate that the sulcal patterns are stable during development (Cachia et al., 2016; Tissier et al., 2018; Figure 2).

### Factors Contributing to the Cortical Folding Process

Cortical folding is a very complex process, involving factors at different scales; see (Zilles et al., 2013; Ronan and Fletcher, 2015; Borrell, 2018; Kroenke and Bayly, 2018; Foubet et al., 2019) for recent reviews on the phylogenetic, cellular and mechanical factors of the cortical folding process. Briefly, several intermingled factors contribute to the fetal processes that influence the shape of the cerebral cortex, including cortical growth (Kuida et al., 1996; Haydar et al., 1999; Chenn and Walsh, 2002; Toro and Burnod, 2005), differential expansion of superior and inferior cortical layers (Richmann et al., 1975; Kriegstein et al., 2006), apoptosis or programmed cell death (Haydar et al., 1999), differential growth of the cortical mantle relatively to the underlying white matter (Tallinen et al., 2016), transitory compartments such as the subplate (Rana et al., 2019), differential neuropil developments (Llinares-Benadero and Borrell, 2019; Mangin et al., 2019), mechanical constraints (Foubet et al., 2019) along with differential tangential expansion (Ronan et al., 2013) induced by a genetics-based protomap and/or structural connectivity through axonal tension forces (Van Essen, 1997, 2020).

Different hypotheses have been proposed to integrate these different factors within a coherent general theory. For instance, the “axonal tension hypothesis” states that axons under tension pull cortical regions which are strongly connected together and cause folds (Van Essen, 1997, 2020); the “radial gradient hypothesis” states that the increase in expansion of the supra-granular layers relative to the infra-granular layers causes buckling (Richmann et al., 1975); the “differential tangential expansion hypothesis” states that tangential expansion of the cortex causes an increase in tangential pressure which is limited though buckling (Le Gros Clark, 1945; Ronan et al., 2013); see Ronan and Fletcher (2015), for a detailed discussion of the pros and cons for the different theories. These theories, with multiple influences from micro- and to macroscopic levels, still remain a topic of intense debates and experimental/simulation studies.

### How to Measure the Cortex Sulcation?

The measure of the cortex sulcation is a difficult issue because the cortical folds are complex 3D structures that are very variable among individuals (Ono et al., 1990; Petrides, 2018). We provide below a selection of methods that can provide, directly or indirectly, information related to the sulcal patterns.

Several studies of the cortex sulcation focused on the amount of cortex buried into the folds using the gyrification index (GI). Such quantitative measure of the gyrification is indirectly related to some sulcal patterns; for instance, the GI is modulated by the presence vs. absence of a fold or by a continuous vs. interrupted fold. This ratio was initially based on contour lengths measured in 2D sections of the brain (Zilles et al., 1988; Moorhead et al., 2006). The main limitation of this measure is due to its 2D approach that cannot capture all the details of the 3D sulcal morphology. Using automatic segmentation of the cortex (Mangin et al., 2004c) and its folds (Perrot et al., 2011), it is now possible to measure the 3D morphology of the sulci. A 3D version of the GI has been proposed, based on the ratio between the area of the sulcal surface area and the area of the convex envelope of the cortex and completed with regional and local indexes, restricted to some specific lobes or sulci (Cachia et al., 2008). The global GI can also be enriched by quantifying the amplitude of different folding wavelengths in different frequency bands (Germanaud et al., 2014). This approach is of particular interest for the study of development, as there is a correspondence between these bands and primary, secondary and tertiary folds (Dubois et al., 2019). Local sulcation measures, such as local GI, local curvature or fractal measures, have also been proposed (Im et al., 2006; Luders et al., 2006; Pienaar et al., 2008; Fischl, 2012) but their anatomical interpretation is not straightforward. For each sulcus, it is also possible to quantify simple morphometric parameters such as the depth, length or opening of the folds (distance between the two walls of each fold; Mangin et al., 2004b; Alemán-Gómez et al., 2013). More sophisticated features quantifying the complex 3D shape have also been introduced (Mangin et al., 2004a; Sun et al., 2012).

However, the main limitation of all these *quantitative* measures is that they cannot accurately assess the *qualitative* features of the sulcal patterns (Figure 1). In addition, these quantitative measures are state, and not trait, markers of the cortex anatomy and can therefore capture neurodegenerative as well as neurodevelopmental processes. One of the major difficulties of devising qualitative measures of the sulcal patterns is the huge variability of the sulcal patterns (Ono et al., 1990; Petrides, 2018). Large sulci are often interrupted, each sulcus piece being susceptible to connecting with the others in various ways. This recombination process often leads to ambiguous configurations for the usual anatomical nomenclature, which creates difficulties for the morphometric study of sulci. These difficulties have led to propose a generic nomenclature of cortical folding defined at a lower scale level (Mangin et al., 2019). A classical way to perform such qualitative analysis of the sulcal patterns is based on visual inspection, which requires anatomical training by an expert, is time consuming and may suffer from subjective bias. An important perspective is the development of methods for the fully automated recognition of cortical folding patterns (Snyder et al., 2019; Borne et al., 2021). An alternative approach is based on graph-based analysis of the spatial organization of the “sulcal pits” (Im et al., 2011; Takerkart et al., 2017), the local maxima of depth of the cortical surface (Lohmann et al., 2008).

A majority of these methods to visualize and analyze the sulcal morphology are freely available in “Morphologist” toolbox, within BrainVISA software^1^.

## Fetal Foundation of Normal and Pathological Cognition

Several longitudinal studies have reported that small variations of the intrauterine environment, assessed by birth weight, are associated with differences in cognitive abilities after birth (Shenkin et al., 2004; Raznahan et al., 2012; Walhovd et al., 2012). Complementary to such a *global* proxy measure of “uterine optimality” (Raznahan et al., 2012), analysis of the sulcal patterns can provide information on prenatal dispositions to normal and pathological cognitive features *via* the prenatal constraints imposed by the anatomy of some specific cortical regions on cognitive development. As presented below, inter-individual variation in the sulcal patterns can therefore be used to search for prenatal differences.

### Normal (Non-clinical) Condition

Cortical sulcation at birth in preterm has been shown to predict infants’ neurobehavioral development several weeks later (Dubois et al., 2008a; Kersbergen et al., 2016). As detailed below, several studies analyzed the sulcal patterns of typically developed participants to investigate the long-term influence of fetal development on cognition.

#### Cognitive Control

Cognitive control (CC), also referred as executive control or self-regulation, including inhibitory control—i.e., the ability to overcome conflicts and inhibit a dominant response—is one of the core executive functions that enables us to resist temptations, automatisms or distractions, habits or interference and allows us to adapt to complex situations using mental flexibility, i.e., dynamic activation/inhibition of competing cognitive strategies (Petersen and Posner, 2012; Diamond, 2013). It plays an important role in academic (Borst et al., 2015) and professional success (Moffitt et al., 2011). It is also involved in the pathophysiology of numerous psychiatric disorders (Diamond, 2013).

A central region of the CC network is the dorsal anterior cingulate cortex (ACC; Petersen and Posner, 2012). The ACC can have two qualitatively distinct sulcal patterns: a “single” type (only the cingulate sulcus is present) and a “double parallel” type (a paracingulate sulcus, PCS, runs parallel to the cingulate sulcus). The PCS is a complex structure that lies dorsal to the cingulate sulcus, found only in humans and chimpanzees (Amiez et al., 2019) with high inter-individual and inter-hemispheric variability (Paus et al., 1996). The infralimbic sulcus is an additional variation; when present (that is, in only about 5% of hemispheres), it runs between the cingulate sulcus and the supracallosal sulcus (Paus et al., 1996).

In adults, asymmetry in the ACC sulcal pattern (i.e., the “double parallel” type with a PCS in one hemisphere and the “single” type in the other hemisphere) is associated with increased CC efficiency at behavioral (Fornito et al., 2004; Huster et al., 2011) and electrophysiological (Huster et al., 2009, 2012) levels. Similar behavioral findings were found during development; an asymmetrical ACC sulcal pattern is associated with increased cognitive control in children at age 5 (Cachia et al., 2014) and 4 years later (Borst et al., 2014). Asymmetry effect on CC was also found for the inferior frontal cortex (IFC), another key region of CC neural network (Tissier et al., 2018). These early neurodevelopmental constraints on later cognitive efficacy are not fixed nor deterministic. Indeed, only a part (~15–20%) of cognitive variability is explained by the sulcal pattern variability (Borst et al., 2014; Cachia et al., 2014; Tissier et al., 2018). These additive effects of ACC and IFC sulcal patterns suggest that distinct early neurodevelopmental mechanisms, involving different brain regions, may contribute to CC. Such interpretation shares analogies with the “common variant-small effect” model in genetics, which posits that frequent genetic polymorphisms have small effects but collectively account for a large portion of the variance. Similarly, each sulcal polymorphism has a small additive effect: ACC and IFC sulcal patterns explained 14% and 3% of the variance of the cognitive scores (of note, effects in genetics are lower since a single genetic variant with a 3%–14% effect would be considered a very large effect). In addition, similarly to epigenetics, different environmental backgrounds, either after birth such as bilingualism (Cachia et al., 2017; Del Maschio et al., 2019) or before birth such as twin pregnancy (Amiez et al., 2018), can modulate the effect of the sulcal pattern on cognition. Interactions between sulcal patterns, similar to epistasis in genetics, have not (yet) been reported.

The ACC is involved in CC but also in other cognitive functions, such as the reality monitoring (Metzak et al., 2015; Simons et al., 2017) and the temperament (Whittle et al., 2009). Analysis of ACC sulcal pattern revealed that absence of the PCS in both left and right hemispheres is associated with lower reality monitoring, i.e., the ability to distinguish information that was generated by internal cognitive functions (e.g., imagination and thought) from information that was derived from the outside world (Buda et al., 2011). In addition, a leftward asymmetric pattern was found to be associated with increased temperamental effortful control and decreased negative affectivity than a rightward pattern (Whittle et al., 2009). This effect was found only for males. In both females and males, a symmetric pattern was associated with increased temperamental affiliation compared to rightward asymmetric ACC sulcal pattern.

#### Academic Abilities

Sulcal studies also revealed that academic abilities requiring intensive learning and training, such as numeracy or literacy, can also be traced back to fetal life. Indeed, the pattern (interrupted or continuous sulcus) of the posterior part of the left lateral occipito-temporal sulcus (OTS), which hosts the visual word form area (VWFA), predicts reading abilities in 10-year-old children (Borst et al., 2016) and also in adults (Cachia et al., 2018). The position of the sulcal interruption of the OTS plays a critical role since only interruption located in the posterior part of the OTS, hosting the VWFA, but not its anterior part, affects reading fluency (Cachia et al., 2018). Comparison of adults who learned to read during adulthood (ex-illiterates) and adults who learned to read during childhood (literates) revealed that age of reading acquisition modulates the effect of OTS sulcal pattern on reading abilities: interruption of the posterior left lateral OTS affected reading abilities in literates but not in ex-illiterates (Cachia et al., 2018). As for cognitive control, these early neurodevelopmental constraints on later cognitive efficacy are not fixed nor deterministic since the effects of the OTS sulcal pattern accounted for ~5% of the variability in reading fluency, as compared to ~65% for environmental factors such as socio-economic status. In children with developmental dyslexia, the sulcal pattern in left parieto-temporal and occipito-temporal regions is not typical (more sulcal pits basins of smaller size) and correlates with reduced reading performance (Im et al., 2016); of note, non-typical sulcal pattern was also found in pre-readers and beginning readers (preschoolers/kindergarteners) with a familial risk of developmental dyslexia.

Regarding numeracy, the absence or presence of branches sectioning the horizontal branch of the intra-parietal sulcus (IPS), a key region for processing numbers, was found to be related to individual differences in math fluency abilities and symbolic number comparison in children and adults (Roell et al., 2021).

### Pathological Conditions

We will detail on this section studies in schizophrenia and affective disorders, two common psychiatric disorders for which clinical and genetic data support early neurodevelopmental deviations (O’shea and Mcinnis, 2016; Murray et al., 2017). A large number of studies analyzed the cortex sulcation in these two disorders in order to investigate the contribution of early neurodevelopment in different clinical features, like symptomatology, age at onset, treatment resistance… We will also report more recent, but less systematic, studies of sulcation in other psychiatric.

### Schizophrenia

Around one century ago, Elmert Ernest Southard visually inspected photos of cerebral cortex in order to investigate the neuropathology of schizophrenia (*dementia praecox*) and found atypical sulcal patterns, in particular in the temporal cortex in patients with hallucinations (Southard, 1915). From visual inspection of clinical anatomical MRI scans, first-episode schizophrenia patients were found to have a reduction in the asymmetry of the lateral sulcus (LS) length, which borders the planum temporal (Hoff et al., 1992). Of note, such atypical LS asymmetry was associated with better cognitive function in patients. Using MR three-dimensional surface rendering and visual classification of the temporal lobe sulcal patterns, schizophrenia patients were found to have a more vertical orientation to the sulci in the temporal lobe in the left hemisphere (Kikinis et al., 1994).

With the development, of computerized brain morphometry methods, several studies in schizophrenia then investigated the sulcation in the whole brain using 2D GI index. An increased amount of buried PFC cortex was found in chronic but also first episode patients with schizophrenia as well as in unaffected siblings and in subjects at high risk of developing schizophrenia (Yücel et al., 2003; Falkai et al., 2006; Harris et al., 2007; Stanfield et al., 2008), suggesting that abnormal PFC folding may be a vulnerability marker to schizophrenia.

Several studies also investigated the sulcal patterns in the PFC, particularly for the orbitofrontal cortex (OFC) and the dorsal anterior cingulate cortex which presents patterns that can be reliably and easily classified from anatomical MRI.

The OFC presents three qualitatively distinct sulcal patterns based on the continuity of the medial orbital sulci (MOS) and lateral orbital sulci (LOS): in Type I, caudal and rostral portions of the LOS are connected, while the MOS are interrupted between caudal and rostral portions of MOS; in Type II, caudal and rostral portions of both the LOS and MOS are connected and continuous LOS and MOS are jointed by the horizontally oriented transverse orbital sulcus (TOS); in Type III, caudal and rostral portions of both LOS and MOS are interrupted (Chiavaras and Petrides, 2000). Unusual sulcal pattern distributions of OFC have been repeatedly reported in patients with established schizophrenia (Nakamura et al., 2007, 2020; Isomura et al., 2017), including in prodromal stage (Nakamura et al., 2019; Takahashi et al., 2019). OFC sulcal pattern is associated with socioeconomic status, cognitive function, symptom severity and impulsivity (Nakamura et al., 2007). These abnormalities are not restricted to schizophrenia but were also reported in other psychiatric conditions (e.g., bipolar disorder, autism spectrum disorder, attention-deficit/hyperactivity disorder, addiction, obsessive-compulsive disorder), suggesting that OFC sulcal pattern is a general transdiagnostic trait marker of brain dysfunction (Patti and Troiani, 2018; Nakamura et al., 2020).

Several studies reported that the sulcal pattern of the ACC shows a notable asymmetry among the general population that is reduced in patients schizophrenia (Yücel et al., 2002; Le Provost et al., 2003; Fornito et al., 2006) and in subjects at high risk to develop a psychosis (Yücel et al., 2003; Wood et al., 2005; Park et al., 2013; Meredith et al., 2014). Reduced ACC asymmetry is associated with lower executive function in patients with schizophrenia (Fornito et al., 2006). In addition, a shorter PCS is associated with a predisposition to hallucinations in patients with schizophrenia (Garrison et al., 2015, 2019; Rollins et al., 2020).

Sulcation impairments in schizophrenia are not limited to the PFC, but also in the Superior Temporal Sulcus (STS). Of note, the STS and PCS overlap in their temporal emergence during the fetal stage: the PCS appears around 30 weeks of gestation (Nishikuni and Ribas, 2013) and the STS forms near 26 weeks (Leroy et al., 2015). In patients with adolescent onset schizophrenia, the collateral sulcus, between the parahippocampal gyrus and the anterior part of the fusiform gyrus, was found shorter compared to typically developing adolescents (Penttila et al., 2008). In patients with auditory verbal hallucinations (AVH), abnormal sulcation have been found in the language-related cortex, including shorter STS (Cachia et al., 2008) and a higher number of duplicated Heshl’s gyrus (Hubl et al., 2010). In addition, in comparison to healthy controls, opposite spatial deviations have been reported in the patients’ right temporo-parietal junction (rTPJ) according to the spatial location of their AVH, i.e., outside head or inside head (Plaze et al., 2011). Besides, self/other attribution of AVH is also associated with the sulcal pattern of the posterior part of the Sylvian fissure, encompassing the TPJ area and the inferior parietal lobule (IPL; Plaze et al., 2015).

Finally, impaired 3D GI was also associated with visual hallucinations (VH), suggesting that VH, and likely the sensory complexity of hallucinations, could be a proxy of the neurodevelopmental weight of schizophrenia (Cachia et al., 2015). In addition, an incomplete hippocampal inversion (Cachia et al., 2020) was also found in patients with VH. Although the literature reports the classical association between VH and neurodegenerative mechanisms, for example in Body-Lewy Dementia or Parkinson’s Disease, these sulcal studies provide the first evidence of an association between neurodevelopmental mechanisms and VH.

### Affective Disorders

Impaired sulcation have also been found in patients with bipolar disorder (BD). Abnormal ACC sulcal pattern was reported in BD, with a decreased frequency of bilateral “double parallel” type in patients (Fornito et al., 2007).

Several studies in BD also extensively analyzed the GI in PFC. Decreased 2D GI in PFC was found in BD patients (McIntosh et al., 2009). Of note, no difference was detected between schizophrenia patients and BD patients (McIntosh et al., 2009) but GI in PFC was correlated with patients’ working memory impairment and IQ, regardless of the diagnostic.

Abnormal GI was also investigated in regard with treatment resistance in BD. Patients with treatment-resistant depression, either bipolar or unipolar, were found to have reduced global 3D GI, while euthymic BD patients did not differ from healthy controls or depressed patients (Penttila et al., 2009b). These findings support the hypothesis that depression that responds particularly poorly to treatment might involve fetal neurodevelopmental factors (Monkul et al., 2005; Ansorge et al., 2007). However, this GI reduction could also be seen as the consequence of a neurodegenerative process (Monkul et al., 2005; Strakowski et al., 2005; Coyle et al., 2006), since quantitative measure of sulcation is also sensitive to atrophy, as well as being a neurodevelopmental measure. Furthermore, in BD patients and healthy subjects, 2D GI in PFC has been shown to significantly decrease with time (Mirakhur et al., 2009). Besides, BD patients with at least one methionine alleles of brain-derived neurotrophic factor (BDNF) showed more important losses in 2D GI, an effect that was associated with gray matter loss in the left hemisphere.

GI in PFC may also provide intermediate phenotype to distinguish subgroup of BD patients, a first step to define genetically more homogenous subtypes of BD. Indeed, studies of the distribution of the age-at-onset support the existence of three subgroups of patients:early-onset (<25 years), intermediate-onset (between 25 and 45 years), and late-onset (>45 years; Leboyer et al., 2005)—with different genetic vulnerability and transmission within families (Grigoroiu-Serbanescu et al., 2001). Intermediate-onset BD patients have lower global 3D GI in the left and right hemispheres and a lower regional 3D GI in the right dorsolateral PFC in comparison to both early-onset patients and healthy subjects (Penttila et al., 2009a). This study was replicated on a large multi-site sample of 263 BD patients and 320 healthy controls (Sarrazin et al., 2018). Early-onset BD patients had an increased 3D regional GI in the right dorsolateral PFC and patients with a positive history of psychosis had a decreased 3D regional GI in the left superior parietal cortex. There was no difference between the whole patient cohort and healthy subjects. These different studies suggest that BD is associated with localized, but not generalized, abnormalities of sulcation, in particular in patients with a heavy neurodevelopmental loading.

### Other Disorders

More recently, deviations of the cortex sulcation have also been found in other disorders (Sasabayashi et al., 2021), but in a less systematic manner.

Hence, in obsessive-compulsive disorder (OCD), impaired sulcal pattern have been reported in OFC (Delahoy et al., 2019) and in ACC (Shim et al., 2009). Impaired OFC has also been found in addiction (Patti et al., 2020) and in autism spectrum disorder (ASD; Watanabe et al., 2014). Patient with ASD also exhibit (poly) microgyria (Piven et al., 1990). Analysis of the sulcal position revealed spatial shifting of the superior and inferior frontal sulci, the superior temporal and the Sylvian fissure (Levitt et al., 2003). More recently, analysis of the perisylvian area revealed that the right anterior caudal ramus of the posterior part of the STS is longer in ASD patients and associated with social cognition deficit (Hotier et al., 2017).

Of note, “extreme” abnormal sulcation can be found in lissencephaly, rare congenital disorders characterized by a smooth cortical surface (Fry et al., 2014). This spectrum of brain malformations, due to the failure of migrating neurons to reach optimal positions in the developing cortex, leads to severe cognitive deficits.

## Discussion

The sulcal patterns offer a window on the potential fetal constraints of the brain on cognitive abilities and clinical symptoms that manifest later in life. Sulcal studies can therefore inform us as to whether individual cognitive or clinical difference is associated in part to preexisting factors related to the structure of the brain defined during the fetal period. Because sulcal patterns are mainly determined before birth and stable across the lifespan (Chi et al., 1977; Cachia et al., 2016; Tissier et al., 2018), findings of sulcal studies suggest a causal role of sulcation in determining later cognitive abilities or impairments. However, a direct causal link has yet to be provided. For instance, for reading abilities (Cachia et al., 2018), longitudinal studies are required to evidence that pre-reading sulcation constrains later reading skill in the same participants, similar to the longitudinal finding that inferotemporal connectivity constrains the VWFA location (Saygin et al., 2016). Regarding the interpretation of sulcal studies, it is important to stress that early neurodevelopmental factors assessed with the cortex sulcation only explain a part of the inter-individual variability. Indeed, other factors, including environmental factors like socio-economic status (SES), schooling, culture, physical activity, stress… also contribute to the cognition and clinical symptoms. For instance, the OTS sulcal pattern explained around 5% of the variability in reading fluency, to be compared to ~65% for SES, SES summarizing several other environmental variables such as presence of duration and quality of schooling, books in the family… (Cachia et al., 2018). Furthermore, beside cumulative effects of sulcation and environmental factors, some evidence suggest possible interactions between sulcation and environmental factors (Gay et al., 2016) that would need to be further investigated. Such interaction between experiential diversity and early neurodevelopment could explain why trauma is critical in some hallucinations, but plays a minor or no role in others (Luhrmann et al., 2019). It also has to be discovered whether cortical sulcation, in addition to its effect on the cognitive efficiency and clinical symptoms, can also modulate the pedagogical and clinical interventions. In the clinical domain, if therapeutic intervention is found to have different effects in patients with different sulcal patterns, it would open new perspectives toward individualized and precision medicine.

**Figure 3:**
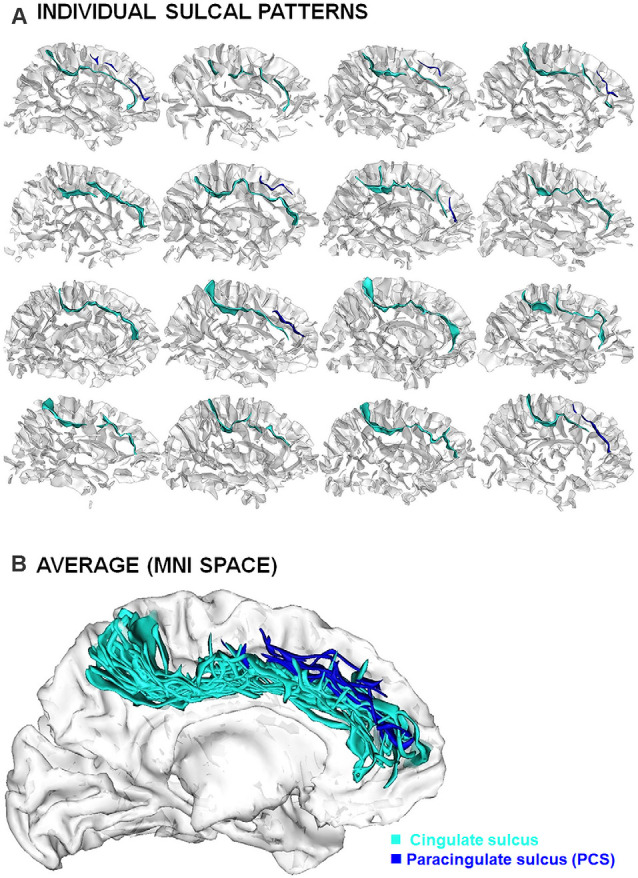
Inter-individual variability of the sulcal patterns. **(A)** Example of individual ACC sulcal patterns in 12 healthy subjects. **(B)** Superimposition of individual sulcal patterns in a common reference space (MNI) after linear spatial normalization.

Sulcal studies have been performed at the group level to investigate general mechanisms of early neurodevelopment on cognition and clinical symptoms. Even though statistically significant findings have been reported, it is important to emphasize the very important variability at individual level. The translation of sulcal findings towards pedagogical or clinical applications, which requires moving from group-level to individual-level, will therefore raise complex methodological issues; see for instance (Duchesnay et al., 2007, 2011). Another critical methodological issue regards the classification of the sulcal pattern that may raise some difficulties due to the very high inter-individual variability (Figure 3; Ono et al., 1990; Petrides, 2018); see for instance the ambiguities in sulcal identification for the ACC region (Leonard et al., 2009). Manual (Garrison, 2017) or semi-automated (Snyder et al., 2019) detailed protocols for the identification and tracing of sulcal pattern have been developed to normalize the process and optimize the inter-rater reliability. A way to overcome sulcal ambiguities is the establishment of a dictionary of the frequent local folding patterns (Sun et al., 2009), for instance based on the “sulcal roots” (Regis et al., 2005), i.e., indivisible and stable sulcal units related to the first folds during fetal life and that can be detected in mature brain from the analysis of the local curvature (Cachia et al., 2003; Mangin et al., 2019) or depth (Yun et al., 2020) of the cortical surface. Another challenging methodological perspective is the development of fully automated techniques for sulcal pattern labeling. The development of such techniques is very complex because of the possible sulcal ambiguities. Recent attempts using cutting-edge machine learning approaches based on Scoring by Non-local Image Patch Estimator (SNIPE), Support Vector Machine (SVM) and 3D Convolution Neural Network (CNN; Borne et al., 2021) along with deep neural networks (Yang et al., 2019) are very encouraging. The development of such “artificial anatomists”, able to automatically identify the sulcal patterns on the whole cortex, will open the possibilities to analyze very large database (e.g., HCP, ABCD, UK Biobank), a first step to crack the “sulcal code”.

## Author Contributions

AC wrote the first draft of the manuscript and created the figures. All authors contributed to the article and approved the submitted version.

## Conflict of Interest

The authors declare that the research was conducted in the absence of any commercial or financial relationships that could be construed as a potential conflict of interest.

## Publisher’s Note

All claims expressed in this article are solely those of the authors and do not necessarily represent those of their affiliated organizations, or those of the publisher, the editors and the reviewers. Any product that may be evaluated in this article, or claim that may be made by its manufacturer, is not guaranteed or endorsed by the publisher.

## Footnotes

^1^ http://brainvisa.info
